# Automatic localization of cochlear implant electrodes using cone beam computed tomography images

**DOI:** 10.1186/s12938-024-01249-5

**Published:** 2024-07-10

**Authors:** Jasmin Thormählen, Benjamin Krüger, Waldo Nogueira

**Affiliations:** 1 Department of Otolaryngology, Hannover Medical School, Karl-Wiechert-Allee 3, 30625 Hannover, Germany; 2 Cluster of Excellence Hearing4all, Hannover, Germany

**Keywords:** Cochlear implant, Computed tomography, Automatic localization, Electrodes

## Abstract

**Background:**

Cochlear implants (CI) are implantable medical devices that enable the perception of sounds and the understanding of speech by electrically stimulating the auditory nerve in case of inner ear damage. The stimulation takes place via an array of electrodes surgically inserted in the cochlea. After CI implantation, cone beam computed tomography (CBCT) is used to evaluate the position of the electrodes. Moreover, CBCT is used in research studies to investigate the relationship between the position of the electrodes and the hearing outcome of CI user. In clinical routine, the estimation of the position of the CI electrodes is done manually, which is very time-consuming.

**Results:**

The aim of this study was to optimize procedures of automatic electrode localization from CBCT data following CI implantation. For this, we analyzed the performance of automatic electrode localization for 150 CBCT data sets of 10 different types of electrode arrays. Our own implementation of the method by Noble and Dawant (Lecture notes in computer science (Including subseries lecture notes in artificial intelligence and lecture notes in bioinformatics), Springer, pp 152–159, 2015. 10.1007/978-3-319-24571-3_19) for automated electrode localization served as a benchmark for evaluation. Differences in the detection rate and the localization accuracy across types of electrode arrays were evaluated and errors were classified. Based on this analysis, we developed a strategy to optimize procedures of automatic electrode localization. It was shown that particularly distantly spaced electrodes in combination with a deep insertion can lead to apical–basal confusions in the localization procedure. This confusion prevents electrodes from being detected or assigned correctly, leading to a deterioration in localization accuracy.

**Conclusions:**

We propose an extended cost function for automatic electrode localization methods that prevents double detection of electrodes to avoid apical–basal confusions. This significantly increased the detection rate by 11.15 percent points and improved the overall localization accuracy by 0.53 mm (1.75 voxels). In comparison to other methods, our proposed cost function does not require any prior knowledge about the individual cochlea anatomy.

## Background

Cochlear implants (CIs) are implantable medical devices that can restore hearing to people suffering from severe to profound sensorineural hearing loss through electrical stimulation of the auditory nerve [1, 2]. The CI consists of two parts, the external and the internal part. The external part consists of a microphone, a sound processor, a battery and a transmitter coil. The internal part, which consists of a receiver coil and an electrode array (EA), is surgically implanted. After CI implantation, in many clinics and hospitals cone beam computed tomography (CBCT) scans are obtained to determine the position of the CI electrodes in the cochlea [3, 4]. The position of the CI electrodes is used to make sure that all electrodes are properly inserted inside the cochlea. Moreover, the position of the CI electrodes may be used in the future to support the programming and fitting of the CI [5–9]. Speech understanding outcomes very widely among CI users and some research findings suggest a relationship between the position of the CI electrodes and the patient's hearing outcome [10–14]. To test this assumption, it is necessary to determine the location of the CI electrodes in the cochlea using CBCT recordings and compare them with the speech understanding results of many subjects.

Determining the location of the CI electrodes in the cochlea is often performed manually, which is very time-consuming. In addition, manual localization requires knowledge of the anatomical structures of the cochlea and can therefore only be performed by a trained expert. In order to determine the positions of the CI electrodes in a large number of CBCT data sets, it is necessary to automate the procedure.

**Fig. 1:**
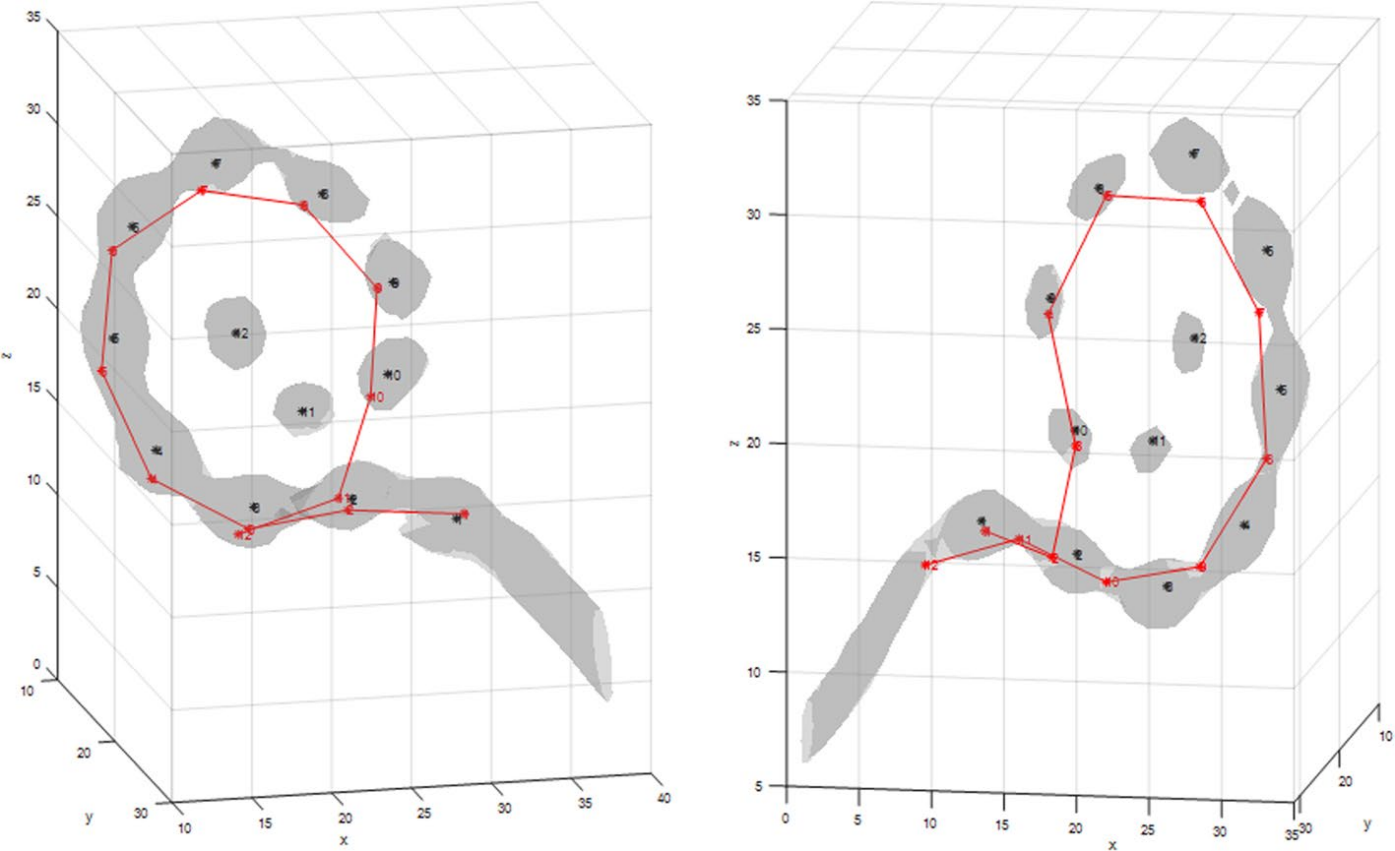
Two examples of apical–basal confusion of the electrode array (EA) Flex 24 from MED-EL. Left: electrodes are detected consecutively in ascending number, from electrode 1 (base) electrode 10 (apex) using an implementation of the algorithm by Noble and Dawant [8]. Next, the algorithm detects electrode number 2 again. Right: after detecting electrode number 2, the algorithm detects electrode number 10. Afterwards, the EA is detected in reverse order

Several approaches for automatic localization of CI electrodes have already been developed [15]. On the one hand, there are approaches that are only suitable for specific EAs. EAs can be classified depending on whether the electrode contacts are closely spaced or distantly spaced. For closely spaced EAs, the individual CI electrodes are not visible on the CBCT image, making it difficult to locate the centers of the individual contacts. For distantly spaced EAs, the contacts are separated from each other so that their centers can be clearly determined. For distantly spaced EAs, one difficulty for the algorithms is to determine how the individual contacts are connected to each other. In a first approach for closely spaced EAs, Zhao et al. [16] proposed to first compute a feature image from the intensity image and the vesselness response of a volume of interest (VOI). Then, using a voxel thinning technique, the medial axis lines for the resulting structures were calculated and the centerline candidates were determined [17]. Then, a cost function was used to find a path with nodes that had the lowest cost. The nodes represent the position of the CI electrodes. Finally, the estimated path was resampled according to the expected electrode spacing. This method was developed only for EAs with closely spaced contacts and is not suitable for other EAs. A second approach by Braithwaite et al. [5] used a defined filter chain (a threshold filter, a spherical filter, and a Gaussian filter) to find the centers of each contact in the CT image. Then, a sorting algorithm was used to arrange the individual contacts in the correct order. If the contacts were too close together, it was not possible to identify the centers of the individual contacts using the filter chain. A first approach suitable for all types of EA used gradient vector flow snakes to locate the 3D centerlines of the EA [9]. A second approach from Noble and Dawant [8], proposed a graph-based method for electrode localization. Here, a midline extraction [17] was used to determine candidate points that could represent possible locations of CI electrodes. Then, using a pathfinding algorithm, a cost function was used to reduce the number of candidate points to match the number of CI electrodes and minimize costs. The path is then refined so that individual nodes can be localized at sub-voxel positions. In Zhao et al. [18], the approach of Noble and Dawant [8] is further developed by the method used to generate candidate points and how these are used in the cost function. To generate the candidate points, the selected volume of interest (VOI) is first scaled up to a size of 0.1 × 0.1 × 0.1 mm^3^. A feature image I_f_ is then calculated. Each feature image is thresholded to 0, creating a region of interest (ROI). Next, a voxel thinning method [17] is applied to the ROIs to generate candidate points. Then, similar to Noble and Dawant [8], a pathfinding algorithm is used to determine the position of the CI electrodes. For this purpose, the found paths are evaluated using a cost function. But unlike Noble and Dawant [8], the shape-based cost function in Zhao et al. [18] takes into account the degree of insertion (DOI) by punishing candidate points that have a smaller DOI value than the last node added into the path as well as the candidate points that are inserted more than half a turn (180°) before or after the endpoint of the path. For this, the DOI is estimated through a registration procedure based on prior knowledge of cochlear anatomy preferably obtained from pre-operative CBCT data. This method has the disadvantage, that it requires manual intervention or the use of additional algorithms for the estimation which increase the complexity of the procedure. Another approach by Bennink et al. [19] used standardized image processing steps to determine the position of the CI electrodes. Here, the CT image is first smoothed with a Gaussian filter. Then, a curve tracing is performed that successively searches for the voxels with the highest intensity within a given area. Finally, the obtained curve is smoothed and correlated with the inter-electrode distances defined in the CI specifications to determine the final electrode position. However, this procedure requires some prior knowledge, e.g., the EA must intersect the left or right boundary of the ROI. In addition, there are concerns that the tracking method of the procedure may fail if the individual contacts are too far apart. Chi et al. [6] proposed a method in which the position of the CI electrodes is determined using a deep learning approach. To do this, a likelihood map is first created in which the values of the voxels are proportional to the distance to the nearest contact. Then, the electrode contacts are determined using a thresholding approach. This approach had the advantage that only the number of electrodes in an EA is required as prior knowledge. However, a direct comparison with Noble and Dawant [8] showed that electrodes could not be successfully located for all datasets examined. In contrast, Noble and Dawant [8] were able to correctly localize the electrodes in all datasets. Moreover, in the cases where localization was successful, the mean localization error of the algorithm by Chi et al. [6] was 0.01 mm better than the algorithm of Noble and Dawant [8]. More recently, an approach based on a Markov random field (MRF) model for electrode localization was proposed by Hachmann and Nogueira [7]. The method aims at minimizing the MRF energy by estimating the maximum a posterior probability (MAP) of the determined EA. In doing so, the method optimizes the localization of all electrodes simultaneously with respect to the distance and angle between each contact. All the mentioned approaches are suitable for all common types of EAs. However, these approaches have weaknesses for distantly space and for deeply inserted EAs. In these cases, confusion often occurs in the assignment of basal and apical electrodes (Fig. 1). This in turn can significantly worsen the localization accuracy. In addition, the presented approaches were all analyzed with only a relatively small CBCT dataset or using specific EAs. In the presented study, we investigate an extension of the localization method of Noble and Dawant [8], representative of a localization algorithm developed for EAs with distantly located electrodes. For this purpose, 150 CBCT data with different EAs were selected and analyzed. Using an own implementation of the reference algorithm by Noble and Dawant [8] resulted in localization errors of the CI electrodes. First, the apical electrodes of an EA were more often not detected. In addition, a confusion between basal and apical electrodes was observed for distantly spaced EAs with an insertion angle larger than 360°. This behavior is illustrated in Fig. 1. The left image shows that the first 10 basal electrodes were correctly recognized. After that, the two basal electrodes, instead of the next apical electrodes, were detected again. In the right image, after detecting the first two basal electrodes, the localization algorithm jumps to the tenth electrode, which is much more apical. Both behaviors of confusion can be explained by the fact that the distance between the individual electrodes and the distance between a basal and an apical electrode is approximately the same due to the helix shape of the cochlea. This leads to a wrong assignment of the individual CI electrodes. This phenomenon does not occur in closely spaced EAs because the electrodes are usually less deeply inserted into the cochlea. Second, even if the electrodes are deeply inserted, the distance between the individual electrodes is smaller than the distance between a basal electrode and an apical electrode at an insertion angle greater than 360°.

The aim of this study is to characterize the apical–basal electrode localization confusion in a newly created dataset containing EAs from different manufacturers and models. Moreover, the current work presents a novel method that can be incorporated in the algorithm of Noble and Dawant [8] and corrects the apical–basal confusion of electrodes without any prior knowledge about the individual cochlea anatomy and does not require any pre-operative CBCT data.

## Results

Three versions of the automatic electrode localization algorithm were evaluated: (1) generalized threshold; (2) individualized threshold; (3) advanced cost function. The algorithm results were evaluated through manual inspection, through correct detection rate and through detection accuracy.

### Detection rate

**Fig. 2:**
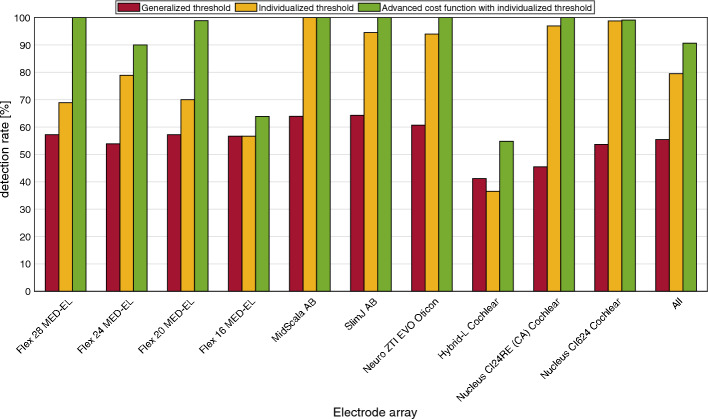
Detection rate for different versions of the automatic electrode localization algorithm. Red is the detection rate for the generalized threshold algorithm, yellow for the individualized threshold algorithm and green is for the advanced cost function with the individualized threshold version of the algorithm

**Fig. 3:**
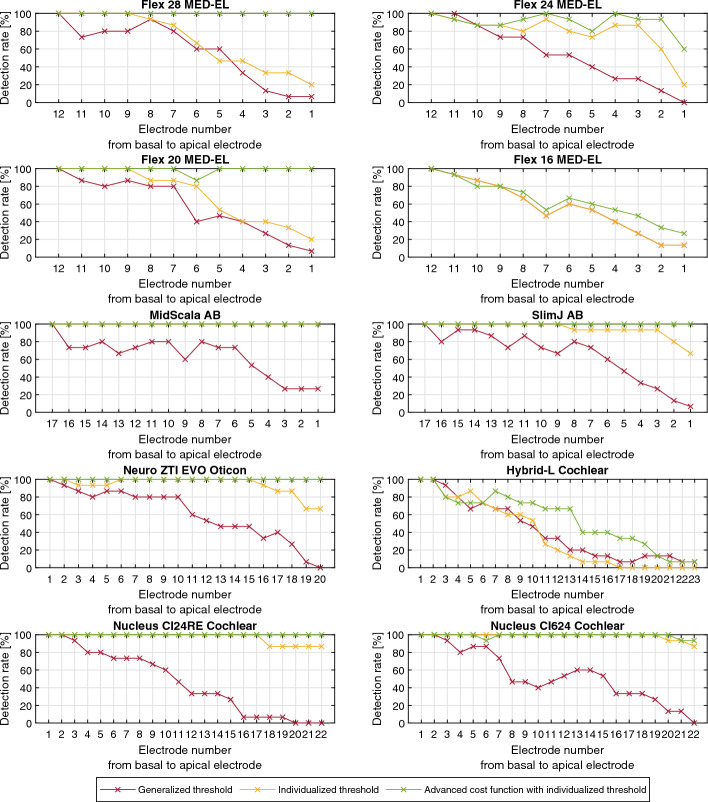
Detection rate for each individual CI electrode and for each electrode array type for different versions of the automatic electrode localization algorithm. Red is the detection rate for the generalized threshold algorithm, yellow for the individualized threshold algorithm and green is for the advanced cost function with the individualized threshold version of the algorithm

Figure 2 shows the average detection rate for the three algorithms (generalized threshold, the individualized threshold and the individualized threshold in combination with the extended cost function) and for each EA type. Figure 3 shows the detection rates obtained for the three conditions obtained for each EA type as a function of the electrode number.

As shown in Fig. 2, the detection rate increased by 24.1 percentage points using individualized thresholds compared to generalized thresholds. An improvement for the individualized thresholds algorithm was observed except for the Hybrid-L and for the Flex 16. For these two datasets, it was noticeable that artifacts were more likely to be detected with the individualized threshold algorithm compared to the generalized threshold algorithm. Moreover, with the Hybrid-L EA from Cochlear, the only threshold with which candidate points could be generated on all CI electrodes was *α*_1_ = 2.37%, therefore, it is only conceivable to increase the detection rate to 100% with the selected threshold value by optimizing the localization process.

One explanation, for the low detection rate of these types of EAs, is the relatively high threshold values of $\alpha_1$ = 2.66% and $\alpha_1$ = 2.37%. This leads to a mean limiting intensity of 1150 HU and 1017 HU. Cortical bone shows an intensity of up to 300 HU in CBCT images [19]. Thus, the filtered image does not only contain artifacts belonging to CI electrodes, but also to the bone. Therefore, candidate points are also generated on bones and causing that more nodes in the path are placed on locations corresponding with artifacts. The largest increase in detection rate was achieved with the Nucleus CI24RE (CA) and Nucleus CI624 EAs (45.15–55.52 percentage points) and the smallest increase with the MED-EL EAs (0–25 percentage points). With the implementation of the extended cost function, the average detection rate could be further increased by 11.2 percentage points compared to the individual threshold algorithm. The detection rate for the MED-EL EAs was particularly increased (between 7.2 and 31.1 percentage points). When looking at the detection rate of the individual electrodes, it can be seen that for many EAs the detection rate was increased specially for the apical electrodes.

A Friedman’s test showed significant differences in detection rate obtained by the general thresholds, the individual thresholds and the individual thresholds combined with the advanced cost function version of the electrode localization algorithm (*p* < 0.001).

Additionally, Dunn–Bonferroni post hoc tests revealed significant differences in the detection rates obtained from all pairwise comparisons: general thresholds and individual thresholds (*p* < 0.001), general thresholds and individual thresholds combined with the advanced cost function (*p* < 0.001), individual thresholds and individual thresholds combined with the advanced cost function (*p* < 0.001). These significance values have been adjusted using the Bonferroni correction for multiple testing.

### Localization accuracy

**Fig. 4:**
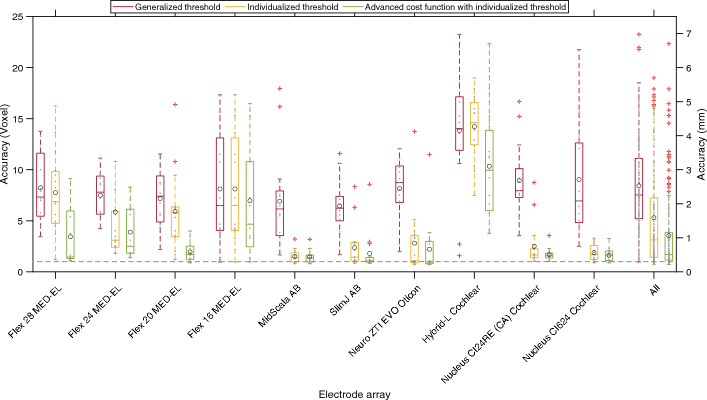
Localization accuracy for all tested electrode arrays (EAs). Red are the results with the generalized threshold algorithm, yellow with the individualized threshold algorithm, and green with the advanced cost function with the individualized threshold algorithm

Figure 4 presents the localization with the generalized threshold algorithm in red, the individualized thresholds algorithm in yellow, and the advanced cost function with individualized threshold in green.

First of all, it can be seen that the localization accuracy with the individualized threshold algorithm is greater than with the generalized threshold algorithm. The exceptions are the EAs Flex 16 from MED-EL, where the localization accuracy with the generalized and individualized threshold algorithms is the same, and the EAs Hybrid-L from Cochlear, where the localization accuracy with the generalized threshold algorithm is better than with the individualized threshold algorithm. Both exceptions can be justified by the same explanation as the one given in the detection rate results. On average, the localization accuracy with the individualized threshold algorithm was 0.95 mm (3.15 voxels) better than with the generalized threshold algorithm. Therefore, the individualized threshold algorithm was used for further investigations in this work. After expanding the cost function, the localization accuracy increased for all EAs. On average, the localization accuracy was improved by 0.53 mm (1.75 voxels), resulting in a localization accuracy of 1.06 mm (3.54 voxels). The increase was strongest for the EAs Flex 28, Flex 24 and Flex 20 from MED-EL and the EA Hybrid-L from Cochlear. In these EAs, due to their large electrode spacing, there was increased confusion between apical–basal electrodes. The results in Fig. 4 indicate that this confusion can be avoided by using the newly proposed advanced cost function algorithm.

A Friedman’s test conducted to determine differences in localization accuracy obtained for general thresholds, individual thresholds and individual thresholds combined with the advanced cost function showed significant differences between conditions (*p* < 0.001).

Supplemented Dunn–Bonferroni post hoc tests revealed significant differences between all pairwise comparisons, general thresholds and individual thresholds (*p* < 0.001), general thresholds and individual thresholds combined with the advanced cost function (*p* < 0.001), individual thresholds and individual thresholds combined with the advanced cost function (*p* < 0.001). Significance values have been adjusted using the Bonferroni correction for multiple testing.

## Discussion

This work deals with the optimization of an automatic localization procedure for the detection of CI electrodes in 150 CBCT images. In general, the localization procedure is based on an own implementation of the algorithm developed by Noble and Dawant [8] as described in Hachmann and Nogueira [7]. In the first step of this work, a dataset containing 150 CBCTs was created. In the next step, the positions of the CI electrodes for the dataset were determined both manually and using the automatic localization algorithm. Then, the errors that occurred when locating the CI electrodes using the algorithm were classified. These errors consist of a double detection of individual electrodes and a confusion in the assignment of apical and basal electrodes. Next, several parameters of the algorithm were adjusted. First, the threshold ${\alpha }_{1}$ was optimized from the cumulative histogram of intensity values across the ROI. For this purpose, threshold values were selected for each EA (individualized algorithm) or a general threshold value across all EAs was used (generalized algorithm). The results showed better localization results for the individualized threshold algorithm. For this reason, the individualized threshold algorithm was used in subsequent optimizations. In a second optimization step, the cost function proposed by Noble and Dawant [8] was extended with the cost term ${C}_{R}$, which should prevent that an electrode is detected twice. This version of the algorithm is termed advanced cost function.

### Dataset

Related work in the area of CI electrode localization used smaller datasets containing fewer CBCTs and less variety of EAs [7, 8, 16]. In our study, we used 150 CBCTs, with ten different EAs resulting in 15 CBCTs datasets per EA. In selecting the EAs, care was taken to ensure that at least one EA was selected from each CI manufacturer. Care was also taken to select both distantly spaced and closely spaced electrode contacts. The new dataset allowed us to investigate the influence of different parameters such as electrode spacing or length of the EA on the localization performance of the automated localization process. For this purpose, a threshold value extracted from the cumulative histogram of intensity values across the ROI was adjusted. Furthermore, additional optimization strategies were developed. The CBCT data were generated with various CT scanners or scanner settings. However, no visual differences in image quality (in terms of contrast or extent of electrode artifact, etc.) were observed during manual labeling by experts. Furthermore, we assume that our method of automatic electrode localization is robust against heterogeneous datasets, with or without our optimization. This is particularly due to the fact that the automatic electrode localization is performed after several post-processing steps, such as thresholding and the use of binary masks, which minimize the effects of different scanners or scanner settings.

### Effect of using advanced parametrization: generalized and individualized threshold approach

When analyzing the localization results with the newly created CBCT dataset, it was found that errors occurred during localization. Double detection of electrodes occurred and confusion of apical and basal electrodes in the localization process was observed. Manual inspection of the results showed that especially the apical electrodes were more difficult to detect. Furthermore, it was observed that there were often no candidate points on the apical electrodes, which are mandatory for the procedure to correctly determine the electrode position. This phenomenon was particularly evident in the MED-EL EAs, where the 5 most apical electrodes had a lower intensity in the CBCT datasets due to their design. To address this error, the threshold *α*_*1*_ of the cumulative histogram was adjusted according to Noble and Dawant [8]. The threshold was chosen so that there was at least one candidate point on each CI electrode and second, a generalized threshold was chosen across all EAs. The results show that with individualized threshold the localization accuracy was 0.95 mm (3.15 voxels) higher and the detection rate was 24.1 percentage points higher than with the generalized threshold. Another phenomenon that occurred more frequently with the generalized threshold approach was that artifacts belonging to bones were incorrectly detected. This could be minimized with the individually adjusted threshold. In addition, with the individualized threshold it was possible to assign at least one candidate point to each CI electrode. This procedure caused that all electrodes could be detected in the correct order with further optimizations.

### Effect of using the advanced cost function

To address the problem of double detection of electrodes, an additional cost factor C_R_ was added to the existing cost function proposed by Noble and Dawant [8]. The mean localization accuracy after optimizing the cost function with the individual threshold was 1.59 mm (5.29 voxels). Compared with the localization accuracy obtained with the individual threshold, the localization accuracy with the new cost factor was increased by 33% to 1.06 mm (3.54 voxels). The average detection rate with the new cost function was 90.67%, improving the detection rate by 11.15 percentage points compared to the individualized threshold algorithm. When manually reviewing the localization results obtained with the advanced cost function algorithm, it was observed that double detection of electrodes occurred only in 3 CBCTs. These CBCTs corresponded with the EA Hybrid-L from Cochlear. Thus, double detection of electrodes was avoided in 98% of the CBCT datasets. However, basal to apical confusion errors still occurred in the localization process.

This is especially important for CIs with a large insertion angle and a large electrode distance, since an apical–basal confusion is more likely to occur. This happens when during the localization process the distance from a basal electrode to an apically located electrode is similar to the inter electrode distance. In addition, the electrode localization accuracy was optimized by setting the threshold of the cumulative histogram such that at least one candidate point lies on each CI electrode.

With our implementation and using the described optimizations, we have examined the localization accuracy when an electrode is detected. The average accuracy is 0.42 mm (1.40 voxels). In comparison the localization accuracy is in average 1.06 mm (3.54 voxels). This shows that when an electrode is detected, it is localized close to the electrodes. The errors that still occur in the localization are related to the correct assignment of the electrodes from the base to the apex.

With our proposed algorithm we are able to determine the positions of the electrodes in the cochlea for different types of EAs using a CBCT, which is not possible for all existing methods [5, 16]. The optimizations made can avoid double detection of CI electrodes in the localization procedure. This is particularly important for EAs with a large insertion angle and a large electrode spacing, as apical–basal confusion occurs here as part of the localization process. Thus, here the distance of a basal electrode to an apically located electrode can be similar to the electrode spacing. The problem of double detection of electrodes was not relevant for the algorithm by Braithwaite et al. [5], because in their algorithm the center of each electrode is determined by a filter chain. When sorting the order of the center points, there can be no double detection, because each electrode is characterized by only one center point.

The issue of electrode misassignment was also considered by Zhao et al. [18]. To avoid localization errors (such as apical–basal confusion), Zhao et al. [18] estimated the DOI. This estimation, in turn, requires prior registration of cochlear anatomy based on, ideally, pre-operative CBCT data. Although their approach is very conclusive, our approach offers some advantages, such as not requiring any image registration which may be computationally expensive.

Our algorithm requires prior knowledge about the number of electrodes, distance between each electrode and the position of the most basal electrode for the localization process. The method of Bennink et al. [19] requires more preconditions such as that the EA must intersect the ROI on the left or right boundary. However, it is equally conceivable that the EA cuts the ROI in front, behind, above or below. However, there are some approaches that do not require prior knowledge of the position of the most basal electrode [5, 8, 16]. The approach of Chi et al. [6] only requires the number of electrodes as prior knowledge. Unlike Chi et al. [6] and other deep learning methods, our approach does not require a training dataset and it is in principle directly applicable to all types of EAs.

In future work, it is conceivable that the extended cost function could be integrated into the algorithm proposed by Hachmann and Nogueira [7] to avoid double detection of electrodes. The algorithm can optimize all electrode positions simultaneously in terms of distances and angles together. For this purpose, an initial path is determined at the beginning of the localization process. This path contains as many nodes as electrodes are expected in the EA. The nodes have a defined distance to each other corresponding with the interelectrode distance. In the course of the localization, this path is then iteratively adapted to the electrode positions. However, in its current status, the algorithm is very computationally and time intensive, which makes it less suitable for the analysis of large data sets. Previous studies show that it is difficult to find a localization procedure that is suitable for all types of EAs [5, 16]. To solve this problem, a newly developed localization method could combine different localization methods: one for widely spaced electrode contacts and one for closely spaced electrode contacts depending on the electrode distance and the resolution of the CBCT scanner.

## Conclusions

This work presented two optimizations of an algorithm to automatically locate CI electrodes in CBCT datasets. For this purpose, a new dataset of 150 CBCT images with 10 different types of EAs were created. In a first optimization of the algorithm, threshold estimation from the cumulative histogram of intensity values in the dataset was adjusted so that there was at least one candidate point on each CI electrode. In a second optimization, a new cost factor was added to the existing cost function of the algorithm. Compared to existing methods, our proposed extension of the cost function enables the minimization of double electrode detections without requiring prior knowledge of the individual cochlear anatomy. The optimizations significantly improved the detection rate by 11.15 percentage points and increased the overall localization accuracy by 0.53 mm (1.75 voxels).

## Methods

### Dataset

The dataset used for evaluation consists of postoperative CBCT scans from 150 implanted CI users including 10 different EAs (15 each) (see Table 1). The dataset was created to analyze the accuracy of the localization algorithm if applied to various EAs differing in the number and size of the electrodes or the spacing between them. The CBCT scans were acquired either with a Xoran Minicat (South State Road, Ann Arbor, United States) or with a Xoran xCat (South State Road, Ann Arbor, United States). The resolution of all CBCT scans was 0.3 × 0.3 × 0.3 mm (except one has the resolution 0.125 × 0.125 × 0.125 mm). The selected dataset contained EAs from the four CI manufactures MED-EL (Innsbruck, Austria), Advanced Bionics (Valencia, CA, Unites States), Oticon Medical (Vallauris, France) and Cochlear (Macquarie Park, NSW, Australia). CBCT scanning was performed postoperatively for all subjects, following the implantation. Tables 2 and 3 provide an overview of the CT scanners and the corresponding settings used for each CI user. The seven most basal electrodes of the MED-EL Flex EA consist of double contacts, and the five most apical are single electrode contacts. For this reason, the CBCT scans resulted in lower intensities for the apical electrodes compared to the basal electrodes. The MidScala and SlimJ EAs from Advanced Bionics have 16 active contacts and one non-stimulating marker contact. For these EAs, the distance between the individual active electrodes is constant and the distance between the most basal electrode and the marker is 3 mm. The Neuro ZTI EVO EA from Oticon Medical has also a constant distance between the electrodes. The EAs from Cochlear contain 22 electrodes. The Hybrid-L EA from Cochlear also features a non-stimulating marker contact.

**Table 1:** Chosen electrode arrays

Name	Manufacturer	Number of electrodes	Electrode spacing (mm)
Flex 28	MED-EL	12	2.1
Flex 24	MED-EL	12	1.9
Flex 20	MED-EL	12	1.5
Flex 16	MED-EL	12	1.0
MidScala	AB	16 + 1	0.975
SlimJ	AB	16 + 1	1.3
Neuro ZTI EVO	Oticon	20	1.2
Hybrid-L	Cochlear	22 + 1	0.6–0.8
Nucleus CI24RE (CA)	Cochlear	22	0.39–0.81
Nucleus CI624	Cochlear	22	0.824–0.95

**Table 2:** Overview of the scan parameters used

CT scanner		KVP (kV)	X-ray tube current (mA)	Exposure time (ms)	Grayscale range (Bit)
MiniCAT	Setting 1	120	7	8400	16
	Setting 2	125	7	8400	16
xCAT	Setting 1	120	6	9600	16
	Setting 2	120	14	9600	16
Morita	Setting 1	90	7	30,800	16

**Table 3:** Overview of which CI user was scanned with which CBCT parameter; NA = not applicable

Electrode type	CT scanner				
	XoranTechnologies				Morita
	MiniCAT		xCAT		
	Setting 1	Setting 2	Setting 1	Setting 2	Setting 1
MED-EL FLEX 28	2–10	NA	NA	1,11–15	NA
MED-EL FLEX 24	NA	1,4,5,8,9,10	2,3,6,7,11–15	NA	NA
MED-EL FLEX 20	3	4–6,8–10	7,11–15	1,2	NA
MED-EL FLEX 16	9	1–5,7,10	6,8,11–14	NA	15
AB MidScala	6	7,10,13,15	8,9,14	1–5,11,12	NA
AB SlimJ	2–4,7,8	9,10	12–15	1,5,6,11	NA
Oticon Neuro ZTI EVO	NA	8	1–7,10–15	NA	NA
Cochlear Hybrid-L	NA	1–3,6,9,10,12	4,5,7,8,11,13–15	NA	NA
Cochlear Nucleus CI24RE	NA	7,8,13,15	1–6,9–14	NA	NA
Cochlear Nucleus CI624	1–7,13	NA	NA	8–12,15	NA

For the entire dataset, a clinical expert determined the electrode locations manually in two different ways. For distantly spaced electrodes, locations were determined labeling the center of the individual electrodes. The location of closely spaced electrodes was determined using a spline that was fitted through the EA. Individual electrode locations were determined using the known geometry of the EA according to Krüger et al. [20]. The manually determined electrode locations were used as ground truth (GT).

The dataset was used to evaluate the improvement that the extension of the cost function we proposed has on the detection rate and localization accuracy.

### Evaluation criteria

The localization accuracy $L_{\text{a}}$ and the detection rate were used to evaluate the automatic electrode localization algorithm. The localization accuracy is based on the averaged localization error defined as the Euclidean distance between a predicted electrode ${\text{el}}_{{\text{pd}},N}$ and the GT electrode position ${\text{el}}_{{\text{GT}},N}$ with the same label or electrode number *N* (see Eq. 1):

1 $$L_{\text{a}} = \mathop \sum \limits_{i = 1}^N \frac{{\left\| {{\text{el}}_{{\text{pd}},N_i } - {\text{el}}_{{\text{GT}},N_i } } \right\|_2 }}{N}.$$

The detection rate was defined as the number of correctly detected electrodes ${\text{el}}_{{\text{detect}}}$ divided by the total number of electrodes ${\text{el}}_i$ in the EA (Eq. 2). An electrode is correctly detected if the Euclidian distance between the location of the automatically predicted electrode ${\text{el}}_{{\text{pd}}}$ and the location of the GT electrode ${\text{el}}_{{\text{GT}}}$ was less than or equal to 0.9 mm (3 voxels):

2 $${\text{Detection}}\,{\text{rate}} = \frac{{\sum {{\text{el}}_{{\text{detect}}} } }}{{\sum {{\text{el}}_i } }}*100,$$

3 $${\text{el}}_{{\text{detect}}} = \left\{ {\begin{array}{*{20}l} {\left\| {{\text{el}}_{{\text{GT}}} - {\text{el}}_{{\text{pd}}} } \right\|_2 \le 0.9\,{\text{mm}}} \hfill & {1\,({\text{detected}})} \hfill \\ {{\text{else}}} \hfill & {0\,({\text{not}}\,{\text{detected}})} \hfill \\ \end{array} } \right.$$

### Graph-based algorithm to locate cochlear implant electrodes

**Fig. 5:**
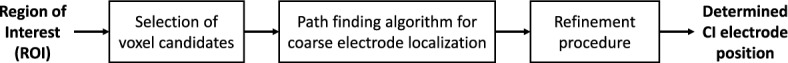
Block diagram illustrating the processing steps to determine the cochlear implant electrode position. This process follows the same structure as Noble and Dawant [8] and Hachmann and Nogueira [7]

In the present study, automated electrode localization was performed using an implementation of the graph-based procedure according to Hachmann and Nogueira [7] which can be considered own implementation of Noble and Dawant [8]. Figure 5 illustrates the processing steps of the algorithm as a block diagram. It takes as input a region of interest (ROI). First, candidate voxels representing potential electrode locations are selected. The next steps consist of a path finding algorithm for a rough electrode localization and a refinement procedure enabling sub-voxel electrode localization.

The input for the localization procedure is a ROI obtained from CBCT data containing the electrode contacts. The ROI was determined based on the GT coordinates. The automatic segmentation of the ROI needs to be implemented in future versions of the algorithm. For this purpose, based on the GT coordinates, a region was defined around the EA with a minimum distance of 3 mm (10 voxels) to the electrodes. Next, a binary mask was generated from the ROI defining a threshold. For this purpose, the cumulative histogram of the intensity values of the voxels contained in the ROI were calculated to define a threshold that depends on the ROI’s dynamic range. According to Noble and Dawant [8], 0.08% of the maximum value of the ROI’s cumulative histogram can be used as threshold *α*_*1*_. From the contours of the resulting binary mask, centerlines were determined whose voxel coordinates served as candidate points for possible electrode locations [17]. From the candidate points, the path finding algorithm generated all possible paths *p* consisting of *L* nodes. The number of nodes (*L*) corresponds to the number of electrodes in the EA. In contrast to Noble and Dawant [8], we used the first node given by the GT corresponding to the most basal electrode location to reduce computing time. According to Hachmann and Nogueira [7], the most basal GT coordinate serves as a reference for the seed node of the path finding algorithm. This significantly shortens the evaluation time while having minimal impact on the determination of localization accuracy [7]. Paths were built up starting from the seed node as first path node p_1_. These paths were evaluated using an initial cost function as shown in Eq. (4):

4 $${{\varvec{Cost}}}_{{{\varvec{initial}}}} \left( {{{\varvec{c}}},{{\varvec{p}}}} \right) = {{\varvec{C}}}_{\text{I}} \left( {{{\varvec{c}}},{{\varvec{p}}}} \right) + {{\varvec{C}}}_{{{\varvec{S}}},{{\varvec{initial}}}} \left( {{{\varvec{c}}},{{\varvec{p}}}} \right).$$

The initial cost function Cost_initial_ consists of an intensity-based *C*_I_ and an initial shape-based *C*_S,initial_ component, where c are the selected candidate points and p are the points already located in the path, which in the case of the initial cost function, was the initial seed point. The intensity-based component is calculated as described below (see Eq. (5)):

5 $$C_{\text{I}} \left( {c,p} \right) = \frac{{\left( {I_{{\text{max}}} - I \left( c \right)} \right)}}{2000} \cdot \left\{ {\begin{array}{*{20}l} {\alpha_3 } \hfill & {i \ge \alpha_4 } \hfill \\ 1 \hfill & {{\text{else}}} \hfill \\ \end{array} } \right..$$

In Eq. (5), *I*_max_ is the maximum intensity of the ROI, *I*(c) is the intensity of child node *c*, *i* is the length of the path *p*. *α*_3_ and *α*_4_ are set to 0.1 and 14. The initial shape-based component is calculated according to Eq. (6)):

6 $${{\varvec{C}}}_{{\bf{S}},{{\varvec{initial}}}} \left( {{{\varvec{c}}},{{\varvec{p}}}} \right) = \left\| {{{\varvec{c}}} - {{\varvec{p}}}_1 } \right\|_2 - {{\varvec{d}}}_1 .$$

The next step is to sort out paths in which the two nodes in the path do not have the desired distance to each other. The desired distance is obtained from the inter electrode spacing defined by the manufactured specifications. Further child nodes c are added to the path as path node *p*_*i*+1_ if located within a certain distance from the path as nodes *p*_*i*_ (Eq. 7):

7 $$\frac{{{{\varvec{d}}}_{{\varvec{i}}} }}{2} < \left\| {{{\varvec{p}}}_{{\varvec{i}}} - {{\varvec{c}}}} \right\|_2 < 2 \cdot {{\varvec{d}}}_{{\varvec{i}}} .$$

In Eq. (7), *d*_*i*_ is the distance between electrodes with indices *i* and *i* + 1 counted from apex to base. In the equation $\left\| {{{\varvec{p}}}_{{\varvec{i}}} - {{\varvec{c}}}} \right\|_2$ describes the Euclidean distance between two vector points, which is also called norm 2. This is defined as follows: $\left\| x \right\|_2 = \sqrt {x_1^2 + x_2^2 + x_x^2 }$, with $x = p_i - c$. Furthermore, a child node should not already exist in the path. The cost of each possible child node is calculated using the cost function shown in Eq. (8). This cost function consists of an intensity-based $C_{\text{I}}$ and a shape-based part $C_{\text{S}}$. Equation (8) shows the calculation of intensity-based costs:

8 $${\text{Cost}}_1 \left( {c,p} \right) = C_{\text{I}} \left( {c,p} \right) + C_{\text{S}} \left( {c,p} \right).$$

In the intensity-based component, child nodes that correspond to a high intensity in the ROI are preferred. For the last $L + 1 - \alpha_4$ electrodes, this cost term is reduced because apical electrodes are less bright in the CBCT image. Equation (9) shows the calculation of the cost of the shape-based part C_S_:

9 $$C_{\text{s}} \left( {c,p} \right) = \alpha_5 - \left( {1 - \left\{ {\begin{array}{*{20}c} {Cos\left( {c,p} \right)} & {Cos\left( {c,p} \right) < 0.5} \\ 1 & {else} \\ \end{array} } \right.} \right) + \left\{ {\begin{array}{*{20}c} { - \alpha_6 Dst \left( {c,p} \right)} & {Dst \left( {c,p} \right) < 0} \\ {\alpha_7 Dst \left( {c,p} \right)} & {else} \\ \end{array} } \right.,$$

10 $${{\varvec{Cos}}}\left( {{{\varvec{c}}},{{\varvec{p}}}} \right) = \frac{{\left( {{{\varvec{c}}} - {\varvec{ p}}_{{\varvec{i}}} } \right)\left( {{{\varvec{p}}}_{{\varvec{i}}} - {\varvec{ p}}_{{{\varvec{i}}} - 1} } \right)}}{{\left\| {{{\varvec{c}}} - {{\varvec{p}}}_{{\varvec{i}}} } \right\|\left\| {{{\varvec{p}}}_{{\varvec{i}}} - {\varvec{ p}}_{{{\varvec{i}}} - 1} } \right\|}};\quad {{\varvec{Dst}}}\left( {{{\varvec{c}}},{{\varvec{p}}}} \right) = \left\| {{\varvec{ c}} - {\varvec{ p}}_{{\varvec{i}}} } \right\|_2 - {\varvec{ d}}_{{\varvec{i}}} .$$

According to Noble and Dawant [8], the constants in Eq. (9) were set to *α*_*5*_ = 1.0, *α*_*6*_ = 5.2 and *α*_*7*_ = 2.0. The first part of the shape-based cost function *C*_*s*_ was a smoothing term that incurs a high cost if adding a child node would result in a sharp bend in the EA. The second part is a distance term that rejects child nodes that do not have the expected distance $d_i$ to the last node in path *p*. The calculation of the cost is performed for all possible child nodes and is added to the already existing costs, from the previous iterations (see Eqs. 11, 12):

11 $${\text{Cost}}_{1,k} = {\text{Cost}}_{1, k - 1} + {\text{Cost}}_1 ,$$

where

12 $$k = 1, \ldots ,L.$$

After this the *P* = 10 paths with lowest cost are saved. This is repeated until the number of nodes *L* in the path is reached. The path with the lowest cost is selected as the optimal path.

In the last step, the optimal path is refined so that the position of each node can be estimated on sub-voxel positions. First, a rectangular grid is placed around each point of ***L***,$\left\{ {{{\varvec{l}}}_{{\varvec{i}}} } \right\}_{i = 1}^{{\varvec{L}}}$ to define node points, which are sampled by $\left\{ {{\varvec{n}}} \right\}^i = \left\{ {{{\varvec{l}}}_i + \alpha_8 \left[ {x, y, z} \right]} \right\}_{x, y, z \in \left[ { - \alpha_9 ,\alpha_9 } \right]}$. Here, $\alpha_8$ and $\alpha_9$ are defined to be 0.12 and 3 mm, respectively. This step aims at refining each estimated position ${{\varvec{l}}}_i$ of every *i*th electrode with a nearby candidate $\left\{ {{\varvec{n}}} \right\}^i$. The cost is thereby determined by means of a second cost function (see Eq. (13)):

13 $${\text{Cost}}_2 \left( {c,p} \right) = - G_\sigma \left( {I\left( c \right)} \right) + \left\{ {\begin{array}{*{20}c} { - \alpha_{10} Dst(c,p)} & {Dst\left( {c,p} \right) < 0} \\ {\alpha_{11} Dst(c,p)} & {else} \\ \end{array} } \right..$$

Here, $G_\sigma \left( {I\left( {{\varvec{c}}} \right)} \right)$ corresponds to the Gaussian filter response of the image with $\sigma = 0.3\,{\text{mm}}$. $\alpha_{10}$ and $\alpha_{11}$ are set to 50 and 20, respectively. The first term of the second cost function is a scaled blob finding filter. The second term penalizes child nodes ***c*** that are not within the expected distance from the last found path electrode ***p***_*i*_. The path finding process is the same as the previous localization step.

### New advances and parametrization for automatic localization of cochlear implant electrodes

**Fig. 6:**
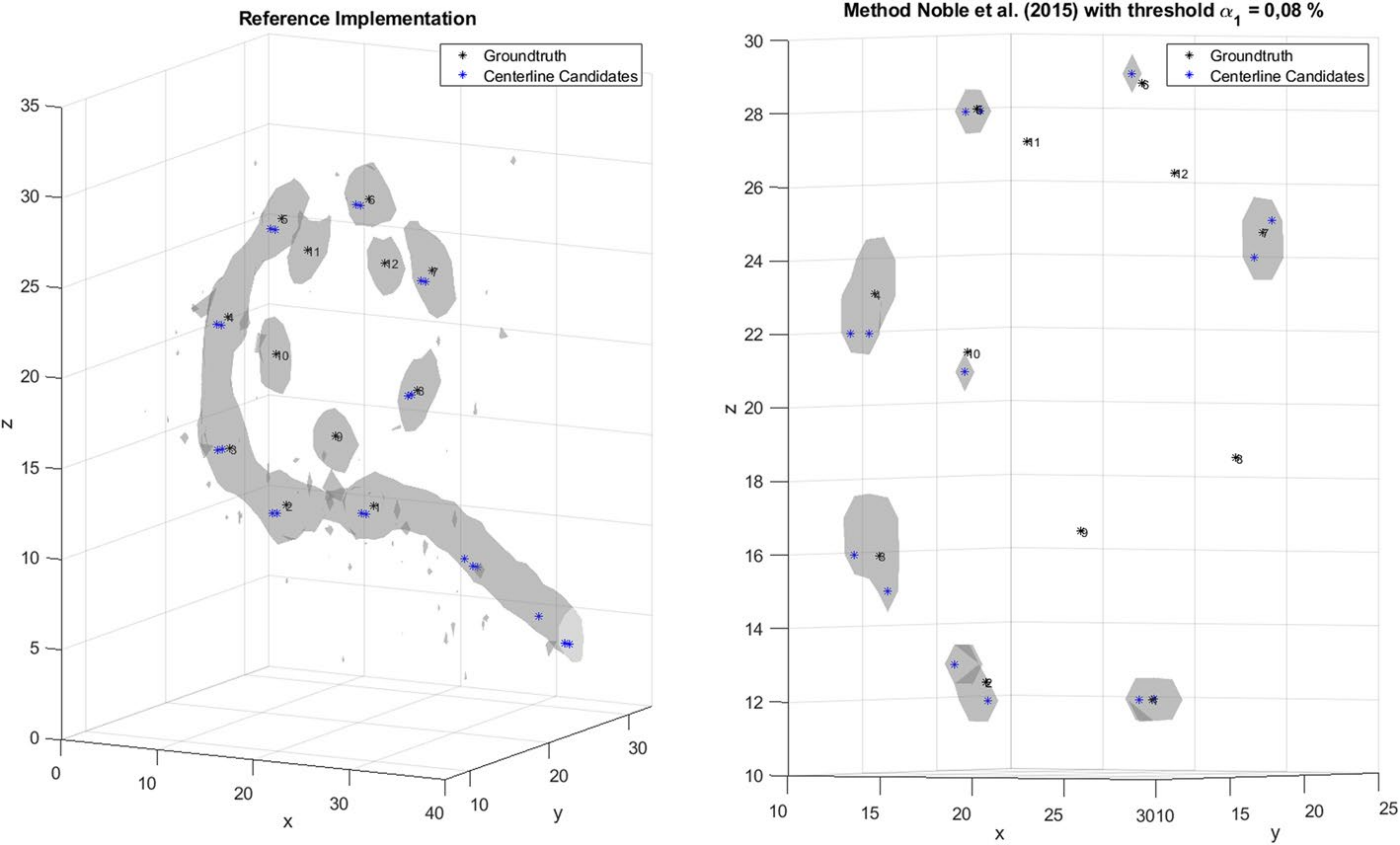
Generated candidate points for an example of the MED-EL's electrode array Flex 28 for the method according to Noble and Dawant [8] with a threshold *α*_1_ of 0.08% (right)

As reported by Hachmann and Nogueira [7], the Noble and Dawant [8] method was not able to correctly detect the position of all electrodes. First, it was found that double detection of the most basal electrode occurred at the beginning of the localization process. Furthermore, some electrodes were detected twice. In the present study, the localization accuracy of the method was investigated with different EAs. The decrease in the detection rate for the apical electrodes can be explained firstly by the nature of the pathfinding method used by Noble and Dawant [8]. The method adds a new node to the path at each iteration in the pathfinding process. This new node must satisfy certain constraints and is expected to minimize the cost function. However, some nodes are added to the path that do not match the desired position of a CI electrode. If a node is detected incorrectly, there is a high probability that subsequent nodes will also be detected incorrectly. Thus, the probability of correctly detecting an electrode at the beginning is higher than at the end of the search path, procedure last nodes in the path correspond to the apical electrodes. Furthermore, in our dataset it was found that where the often no candidate points are placed on the apical electrodes, which is a prerequisite to localize them (see Fig. 6, left). This effect was particularly clear for MED-EL EAs, because the apical electrodes have lower intensity in the CBCT image due to their design.

In our implementation of the algorithm by Noble and Dawant [8], the extraction of the centerlines from the binary mask was performed differently. It was analyzed whether it is possible to generate at least one candidate point on all electrodes with a threshold value proposed by Noble from the cumulative histogram. The threshold was set to *α*_*1*_ = 0.08% as in Noble and Dawant [8]. As observed in Fig. 6 (right), no candidate points were generated on the apical electrodes. Moreover, it can be seen in Fig. 6 (right) that no artifact can be seen on CI electrodes 8,9,11 and 12. This means that the threshold derived from the cumulative histogram caused removal of relevant information from the ROI required for the localization of electrodes in EAs of MED-EL.

Therefore, it was necessary to re-parameterize the threshold from the cumulative histogram for each EA type. The aim was to select the threshold that maximized the likelihood that each CI electrode gets assigned at least one candidate point.

**Fig. 7:**
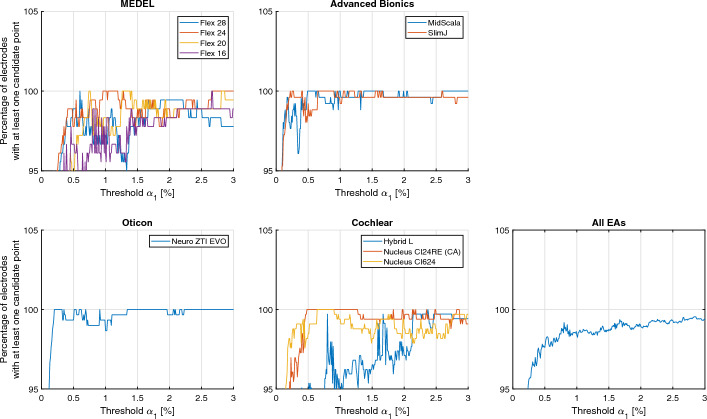
Percentage of electrodes with at least one centerline candidate is allocated for each electrode array type. The right most subplot presents the average across each type of electrode array

To ensure this, the manually determined electrode positions are used for the determination. To parameterize the threshold *α*_*1*_, a range of values from 0.01 to 3% with a step size of 0.01% was investigated. This range was chosen so that there was a threshold for which all CI electrodes were included in the ROI and there were hardly any disturbing artifacts in the ROI. For each threshold, Fig. 7 shows how many electrodes have at least one candidate point within the radius of the GT position, where the radius is 0.9 mm (3 pixels). Table 4 shows the thresholds for each EA and the averaged thresholds across EAs.

**Table 4:** Individual threshold derived from the cumulative histogram for each electrode array (EA) and average across EAs

EA	Customer	Threshold ${\alpha }_{1}$ (%)
Flex 28	MED-EL	0.60
Flex 24	MED-EL	0.96
Flex 20	MED-EL	0.74
Flex 16	MED-EL	2.66
MidScala	AB	0.49
SlimJ	AB	0.22
Neuro ZTI EVO	Oticon	0.20
Hybrid-L	Cochlear	2.37
Nucleus CI24RE (CA)	Cochlear	0.48
Nucleus CI624	Cochlear	0.64
Generalized Threshold	–	2.66

### Advanced cost function

For large insertion angles, the apical electrodes are close to the basal electrodes with a distance that is in the same range as the electrode spacing of the neighboring electrodes or inter-electrode distance. Paths that cross the EA from the apical to the basal side or vice versa might have a lower cost than pathways that arrange the electrodes in the correct order. In addition, Hachmann and Nogueira [7] reported that in the procedure of Noble and Dawant [8], the most basal electrode is often detected twice. Therefore, we proposed an extended cost function to prevent this behavior as follows:

14 $${\text{Cost}}_{{\text{adv}}{.}} = C_{\text{I}} \left( {c,p} \right) + C_{\text{S}} \left( {c,p} \right) + C_{\text{R}} \left( {c,p} \right),$$

15 $$C_{\text{R}} = \left\{ {\begin{array}{*{20}c} 0 & {{\text{diff}}_{{\text{min}}} > \alpha_{12} } \\ \infty & {{\text{else}}} \\ \end{array} } \right..$$

*C*_*R*_ (redetection of CI electrodes) is the new extended cost term. To determine this cost term *C*_*R*_, the Euclidean distances from a candidate point *c* to each node already found in the path p are first calculated. Then, the smallest difference diff_min_ between the candidate point *c* and a path node *p*_*i*_ is determined. This could be expressed mathematically as ${\text{diff}}_{{\text{min}}} = {\mathop {\min }\limits_{{\phantom{a}}}} ({\text{diff}})$. Now diff_min_ is compared to the defined threshold value *α*_12_, which describes the limit range for which no further child node c will be added to the path around a node found in the path. The threshold *α*_12_ is set to 2/3 of the electrode spacing defined in the EA specification. If diff_min_ is less than *α*_12_, a cost of infinity is assigned to *C*_R_, and if diff_min_ is greater than this threshold a cost of 0 is assigned. The new cost factor *C*_*R*_ is integrated as a summand into the existing cost function of Noble and Dawant [8] as shown in Eq. (14).

### Analysis of errors made by the algorithm to automatically localize cochlear implant electrodes

In the first step of the analysis, the results of the automatic localization algorithm were manually analyzed. In this way, errors that occurred during the automatic localization process can be classified. Table 5 shows these errors in percentage.

**Table 5:** Manually analyzed detected errors for three versions of the algorithm: generalized threshold, individualized threshold and individualized threshold with advanced cost function

Electrode array	Generalized threshold			Individualized threshold			Individualized threshold with advanced cost function		
	Detection of artifacts (%)	Double detection (%)	Confusion a–b (%)	Detection of artifacts (%)	Double detection (%)	Confusion a–b (%)	Detection of artifacts (%)	Double detection (%)	Confusion a–b (%)
Flex 28	33	33	13	0	40	20	0	0	0
Flex 24	87	13	0	20	20	27	27	0	0
Flex 20	53	47	0	0	67	0	7	0	0
Flex 16	67	13	0	67	13	0	67	0	0
MidScala	73	7	0	0	0	0	0	0	0
SlimJ	73	7	0	0	27	7	0	0	0
Neuro ZTI EVO	53	27	7	0	7	7	0	0	0
Hybrid-L	47	33	0	47	47	0	67	20	0
Nucleus CI24RE	7	47	0	7	0	0	7	0	0
Nucleus CI624	73	13	0	13	0	0	13	0	0
Average	55	25	2	15	23	6	19	2	0

Three types of errors are considered: first, the detection of artifacts, where the localization process erroneously includes in the path nodes that corresponds with artifacts. Second, the detection error describing the double detection of CI electrodes. Third, apical and basal (a–b) confusion of electrodes

Three types of errors were considered: first, the electrodes can be detected twice. Second, the apical and basal electrodes can be confused during the detection process, in this case two subtypes of errors can be identified. In the first subtype, an apical–basal (a–b) confusion can occur (see Fig. 1, left). In this case, first basal electrodes are detected correctly, but afterwards it may happen that one of the basal electrodes is detected again. In the second subtype, a basal–apical confusion is considered (see Fig. 1, right). Here, an apical electrode is detected after the first basal electrode and the electrodes are then detected backwards. Third, nodes are incorrectly placed at locations corresponding with artifacts caused by, e.g., bones or wire leads in the localization procedure. Using the generalized threshold algorithm the third type of errors occurs in 55% of the CBCT data sets. This can be explained by the high threshold value used in the generalized threshold algorithm causing that many candidate points are located on artifacts and not on the actual CI electrodes. With the individualized threshold, this error occurs only in 12% of the CBCT data sets. The error is most pronounced with the Flex 16 EAs from MED-EL (67%) and with the Hybrid-L EAs from Cochlear (47%). For both types of EAs, the threshold value was chosen very high compared to the other EAs, generating many candidate points on artifacts. Thus, for this EAs it is more likely that nodes in the path are placed on artifacts and therefore detected as electrodes by the localization procedure. In addition, the Flex 20 EAs from MED-EL, Nucleus CI24RE and Nucleus CI624 also occasionally detected artifacts as nodes in the path. This indicates that the selected threshold also generates candidate points on artifacts. By extending the cost function, the error could not be minimized. This was expected, since the newly proposed advanced cost function does not aim at minimizing the detection of artifacts. However, for some data sets, the number of cases where nodes in the path were located on artifacts increased by 7 percentage points for MED-EL's Flex 24, by 7 percentage points for MED-EL's Flex 20, and by 27 percentage points for Cochlear's Hybrid-L. When comparing the CBCT data sets in which artifact detection occurred and after extending the advanced cost function based on the individual threshold results, it was found that the advanced cost function eliminated localization errors related to double electrode detection, but resulted in artifact detection. On average, localization errors occurred for the generalized threshold and the individualized threshold algorithms for a similar number of data sets. Using the newly proposed advanced cost function, the detection error could be reduced. Errors occurred only in 3 CBCT data sets of the EA Hybrid-L from Cochlear. For these datasets, no improvement in localization could be achieved using the new advanced cost function. Compared to the generalized threshold algorithm, there was an increased confusion of apical–basal electrodes than with the individualized threshold. Using the advanced cost function, the apical–basal confusion were completely eliminated. When considering the data sets where basal apical electrode confusions were observed, no double detection of electrodes occurred. Thus, duplicate detection of electrodes was eliminated by extending the cost function in 98% of the CBCT data sets.

### Statistical analysis

All statistical analyses were performed using the Friedman’s test and Dunn–Bonferroni post hoc tests. A p-value of less than 0.001 was chosen as the level of significance.

## Data Availability

Not applicable.

## References

[1] Lenarz T. Cochlear implant—state of the art. Laryngorhinootologie. 2017;96:S123–51. 10.1055/s-0043-101812. 28499298 10.1055/s-0043-101812

[2] Wilson BS, Dorman MF. Cochlear implants: a remarkable past and a brilliant future. Hear Res. 2008;242:3–21. 10.1016/j.heares.2008.06.005. 18616994 10.1016/j.heares.2008.06.005 PMC3707130

[3] Saeed SR, Selvadurai D, Beale T, Biggs N, Murray B, Gibson P, Risi F, Boyd P. The use of cone-beam computed tomography to determine cochlear implant electrode position in human temporal bones. Otol Neurotol. 2014;35:1338–44. 10.1097/MAO.0000000000000295. 24809280 10.1097/MAO.0000000000000295

[4] Würfel W, Lanfermann H, Lenarz T, Majdani O. Cochlear length determination using Cone Beam Computed Tomography in a clinical setting. Hear Res. 2014;316:65–72. 10.1016/j.heares.2014.07.013. 25124151 10.1016/j.heares.2014.07.013

[5] Braithwaite B, Kjer HM, Fagertun J, Ballester MAG, Dhanasingh A, Mistrik P, Gerber N, Paulsen RR. Cochlear implant electrode localization in post-operative CT using a spherical measure. In: Proceedings of international symposium on biomedical imaging 2016-June. 2016. p. 1329–1333. 10.1109/ISBI.2016.7493512

[6] Chi Y, Wang J, Zhao Y, Noble JH, Dawant BM. A deep-learning-based method for the localization of cochlear implant electrodes in ct images. In: Proceedings of international symposium on biomedical imaging 2019-April. 2019. p. 1141–1145. 10.1109/ISBI.2019.8759536

[7] Hachmann H, Nogueira W. Localization of cochlear implant electrodes from cone beam computed tomography using particle belief propagation. Benjamin Kr¨ Bodo Rosenhahn Leibniz University Hanover, Germany Department of Otorhinolaryngology, Hannover Medical School, Hanover, Germ. 2021.

[8] Noble JH, Dawant BM. Automatic graph-based localization of cochlear implant electrodes in CT. In: Lecture notes in computer science (Including subseries lecture notes in artificial intelligence and lecture notes in bioinformatics). Springer; 2015. p. 152–159. 10.1007/978-3-319-24571-3_19 10.1007/978-3-319-24571-3_19 PMC4854292 27158686

[9] Noble JH, Schuman TA, Wright CG, Labadie RF, Dawant BM. Automatic identification of cochlear implant electrode arrays for post-operative assessment. Med Imaging 2011 Image Process. 2011;7962:796217. 10.1117/12.878490. 10.1117/12.878490 PMC4450802 26041945

[10] Aschendorff A, Kubalek R, Turowski B, Zanella F, Hochmuth A, Schumacher M, Klenzner T, Laszig R. Quality control after cochlear implant surgery by means of rotational tomography. Otol Neurotol. 2005;26:34–7. 10.1097/00129492-200501000-00007. 15699717 10.1097/00129492-200501000-00007

[11] Skinner MW, Holden TA, Whiting BR, Voie AH, Brunsden B, Neely JG, Saxon EA, Hullar TE, Finley CC. In vivo estimates of the position of advanced bionics electrode arrays in the human cochlea. Ann Otol Rhinol Laryngol. 2007;116:2–24. 10.1177/000348940711600401. 17542465

[12] Verbist BM, Frijns JHM, Geleijns J, Van Buchem MA. Multisection CT as a valuable tool in the postoperative assessment of cochlear implant patients. Am J Neuroradiol. 2005;26:424–9. 15709150 PMC7974105

[13] Wanna GB, Noble JH, Carlson ML, Gifford RH, Dietrich MS, Haynes DS, Dawant BM, Labadie RF. Impact of electrode design and surgical approach on scalar location and cochlear implant outcomes. Laryngoscope. 2014;124:S1–7. 10.1002/lary.24728. 10.1002/lary.24728 PMC4209201 24764083

[14] Wanna GB, Noble JH, McRackan TR, Dawant BM, Dietrich MS, Watkins LD, Rivas A, Schuman TA, Labadie RF. Assessment of electrode placement and audiological outcomes in bilateral cochlear implantation. Otol Neurotol. 2011;32:428–32. 10.1097/MAO.0b013e3182096dc2. 21283037 10.1097/MAO.0b013e3182096dc2 PMC4144165

[15] Sismono F, Leblans M, Mancini L, Veneziano A, Zanini F, Dirckx J, Bernaerts A, de Foer B, Offeciers E, Zarowski A. 3D-localisation of cochlear implant electrode contacts in relation to anatomical structures from in vivo cone-beam computed tomography. Hear Res. 2022. 10.1016/j.heares.2022.108537. 10.1016/j.heares.2022.108537 35672191

[16] Zhao Y, Dawant BM, Labadie RF, Noble JH. Automatic localization of closely spaced cochlear implant electrode arrays in clinical CTs. Med Phys. 2018;45:5030–40. 10.1002/mp.13185. 30218461 10.1002/mp.13185 PMC7185475

[17] Bouix S, Siddiqi K, Tannenbaum A. Flux driven automatic centerline extraction. Med Image Anal. 2005;9:209–21. 10.1016/j.media.2004.06.026. 15854842 10.1016/j.media.2004.06.026

[18] Zhao Y, Chakravorti S, Labadie RF, Dawant BM, Noble JH. Automatic graph-based method for localization of cochlear implant electrode arrays in clinical CT with sub-voxel accuracy. Medical Image Analysis 2019;52:1–12. 10.1016/j.media.2018.11.005 10.1016/j.media.2018.11.005 PMC6543817 30468968

[19] Bennink E, Peters JPM, Wendrich AW, Vonken E, Van Zanten GA, Viergever MA. Automatic localization of cochlear implant electrode contacts in CT. Ear Hear. 2017;38:e376–84. 10.1097/AUD.0000000000000438. 28379904 10.1097/AUD.0000000000000438

[20] Krüger B, Büchner A, Nogueira W. Simultaneous masking between electric and acoustic stimulation in cochlear implant users with residual low-frequency hearing. Hear Res. 2017;353:185–96. 10.1016/j.heares.2017.06.014. 28688755 10.1016/j.heares.2017.06.014

